# Impact of RAV1-engineering on poplar biomass production: a short-rotation coppice field trial

**DOI:** 10.1186/s13068-017-0795-z

**Published:** 2017-05-02

**Authors:** Alicia Moreno-Cortés, José Manuel Ramos-Sánchez, Tamara Hernández-Verdeja, Pablo González-Melendi, Ana Alves, Rita Simões, José Carlos Rodrigues, Mercedes Guijarro, Isabel Canellas, Hortensia Sixto, Isabel Allona

**Affiliations:** 1 Centro de Biotecnología y Genómica de Plantas, Universidad Politécnica de Madrid (UPM)–Instituto Nacional de Investigación y Tecnología Agraria y Alimentaria (INIA), Campus Montegancedo UPM, 28223 Pozuelo de Alarcón, Spain; 2 Departamento de Biotecnología-Biología Vegetal, Escuela Técnica Superior de Ingeniería Agronómica, Alimentaria y de Biosistemas, UPM, 28040 Madrid, Spain; 3 Centro de Estudos Florestais, Instituto Superior de Agronomia, Universidade de Lisboa, Tapada da Ajuda, 1349-017 Lisboa, Portugal; 4 Centro de Investigación Forestal, Instituto Nacional de Investigación y Tecnología Agraria y Alimentaria (INIA), Carretera de la Coruña km 7.5, 28040 Madrid, Spain

**Keywords:** Poplar, Tree biotechnology, RAV1, Sylleptic branching, Sylleptic branchiness, Lignocellulosic biomass, Field trial, Short-rotation coppice (SRC), TEM1

## Abstract

**Background:**

Early branching or syllepsis has been positively correlated with high biomass yields in short-rotation coppice (SRC) poplar plantations, which could represent an important lignocellulosic feedstock for the production of second-generation bioenergy. In prior work, we generated hybrid poplars overexpressing the chestnut gene *RELATED TO ABI3/VP1 1* (*CsRAV1*), which featured c. 80% more sylleptic branches than non-modified trees in growth chambers. Given the high plasticity of syllepsis, we established a field trial to monitor the performance of these trees under outdoor conditions and a SRC management.

**Results:**

We examined two CsRAV1-overexpression poplar events for their ability to maintain syllepsis and their potential to enhance biomass production. Two poplar events with reduced expression of the *CsRAV1* homologous poplar genes *PtaRAV1* and *PtaRAV2* were also included in the trial. Under our culture conditions, CsRAV1-overexpression poplars continued developing syllepsis over two cultivation cycles. Biomass production increased on completion of the first cycle for one of the overexpression events, showing unaltered structural, chemical, or combustion wood properties. On completion of the second cycle, aerial growth and biomass yields of both overexpression events were reduced as compared to the control.

**Conclusions:**

These findings support the potential application of CsRAV1-overexpression to increase syllepsis in commercial elite trees without changing their wood quality. However, the syllepsis triggered by the introduction of this genetic modification appeared not to be sufficient to sustain and enhance biomass production.

**Electronic supplementary material:**

The online version of this article (doi:10.1186/s13068-017-0795-z) contains supplementary material, which is available to authorized users.

## Background

Lignocellulosic biomass production is met with the challenge to enhance yields and improve physical and chemical traits to become a sustainable, carbon–neutral renewable energy source [1, 2]. Energy produced from lignocellulosic crops will help alleviate our current high dependency on fossil fuels and reduce greenhouse gas emissions responsible for global warming. A further benefit is that such crops do not directly compete with food demand [3, 4]. This has sparked a recent interest in short-rotation coppice (SRC) cultivation of fast-growing species such as poplar for the production of lignocellulosic biomass [5]. Coppicing promotes the resprout of multiple shoots, which increases final biomass, and enables multiple harvests from the original rootstock [6, 7]. Growth- and development-related traits are fundamental components of productivity. In poplar, numerous studies have investigated the relative contribution of several of these traits to productivity and their degree of reliability as productivity determinants in field conditions, particularly when poplars are cultivated as SRC [8–10]. Recent advances have been made in the identification of putative loci underlying phenotypic variation of growth- and development -related traits. These works explored natural genetic variation by means of QTLs [11] genome-wide association studies (GWAS), from populations of *Populus* species growing in common gardens [12–14], and as SRC [15].

Among those traits, early or sylleptic branching has been reported to be positively correlated with high biomass yields [16–21]. Trees growing in temperate and boreal regions need to go through a stage of winter dormancy to develop so-called proleptic branches from axillary meristems formed the preceding year. Some poplar species produce early or sylleptic branches without undergoing a dormant period [22]. Syllepsis adds leaf area per se, but also leaves on sylleptic branches are larger and often grow faster than those on the main axis [23]. This additional leaf area helps to rapidly close the canopy, increasing light interception and suppressing weed growth, which is especially important for the establishment of a SRC plantation and biomass production [24, 25]. However, early branching is a highly plastic trait, strongly affected by the availability of resources and environmental cues [17, 19–21, 23, 26, 27]. Actually, sylleptic branches often show a shorter lifespan than proleptics but, in this short time, they play an important role in the carbon balance, providing a quick return for a relatively small resources investment [16]. These features make syllepsis a valuable productivity-related trait with the potential for the development of new high-yielding SRC genotypes [25]. Although in poplar syllepsis shows much genetic variation and high heritability [17, 23], available data regarding the specific loci and mechanisms controlling syllepsis are still limited. It is well established that auxins play a key role in apical dominance and syllepsis in poplar [28, 29]. Hence, genes related to auxins or to hormones affecting auxin signals are targets to optimize branching for biomass production via the release of axillary buds from paradormancy [30, 31]. However, experiences in the field with engineered trees for any of these genes and their impact on biomass yield have not been carried out so far.

In prior work, we generated hybrid poplars overexpressing the chestnut gene *RELATED TO ABI3/VP1 1* (*CsRAV1*) homolog to *TEMPRANILLO 1* and *TEMPRANILLO 2* from Arabidopsis [32]. These trees featured c. 80% more sylleptic branches than non-modified or *PtaRAV1* and *PtaRAV2* downregulated trees in growth chambers, under controlled conditions [33]. Tree performance in a greenhouse in terms of syllepsis or any other trait may significantly differ from the situation outdoors, where trees may show greater phenotypic variation [17, 21, 27, 34]. Therefore, field trials to monitor tree performance under natural conditions over several years are needed to select the best events or individuals [35]. So far, reports of field trials on genetically engineered trees are scarce and, with several exceptions, have mostly pursued lignin modification [36–39]. Here we report a field trial, in which we examined two poplar transgenic events overexpressing CsRAV1. These transgenics were tested for their ability to maintain this trait under field conditions, their wood properties, and their potential to enhance biomass production under SRC. The trial was run for four years, during which two cultivation cycles were conducted. Transgenic poplars showing a reduced expression of endogenous *PtaRAV1* and *PtaRAV2* were also included in the trial.

## Methods

### Field trial design, establishment, and management

A field trial was designed to test the growth performance of transgenic *Populus tremula x P. alba* INRA clone 717 1B hybrid poplars. The trees included were the wild-type genotype as control (WT), events #37 and #60 of transformed trees carrying the *35S::3xHA:CsRAV1* cassette (hereafter referred to as CsRAV1-overexpression or CsRAV1 OX events), and events #1 and #22 of transformed trees carrying the *35S::PtaRAV1*-*hpiRNA* cassette (hereafter referred to as PtaRAV1&2-knockdown or PtaRAV1&2 KD events). CsRAV1-overexpression events #37 and #60 were selected on the basis of their high branch syllepsis of c. 80% shown when growing under controlled environmental conditions. The criterion for the selection of PtaRAV1&2-knockdown events #1 and #22 was their *PtaRAV1* and *PtaRAV2* transcript abundances, lower than in the wild-type genotype [33]. *In vitro*-rooted cuttings were initially potted in March 2012 and grown in the greenhouse as previously described [33]. The field trial was established in July 2012 in an experimental plot in Madrid (Spain) after obtaining a permit for the release of genetically modified higher plants from the Spanish authorities (notification numbers B/ES/12/30 and B/ES/12/34). At that time, plants were four-month old and had reached a height of c. 2 m. After planting, one WT individual died and five PtaRAV1&2-knockdown #22 lost their shoot tips, so they were excluded from the statistical analysis of sylleptic branching the first year. The trial design included 30 individual trees per genotype distributed in 3 blocks of 10 trees each. The experimental plot area was 204 m^2^, and the plantation density was 10,000 trees/ha. Trees were planted in 12 × 17 rows with spacings of 2 × 0.5 m. To avoid edge effects, an additional row around the trial was planted using the genotype I-214 (*P. x canadensis* Moench.). A protective fence (mesh size 4 cm) was installed around the plot to prevent access of *Leporidae*. The trial was run for two cultivation cycles during 4 years: a first cycle from 2012 to 2013, and a second cycle from 2014 to 2015. Given the flowering time of this hybrid poplar of around 4–5 years, the trees did not flower during the trial.

Each year from June to September, the plot was drip-irrigated. At the beginning of each growing season, a complex fertilizer (N21:P8:K11) was applied at a dose of 25 g per tree. Weed spreading was avoided using an anti-weeds cover in the plantation. No herbicides were used. For pest and disease control, the following treatments were applied: 0.04% deltamethrin against *Gypsonoma aceriana* Dupn. (May 2013), 0.06% imidacloprid against *Myzus persicae* (August 2013), and 0.1% abamectin against *Tetranychus urticae* (August 2014).

### Production of antibodies against the poplar RAV1 protein

Polyclonal antibodies were raised against the poplar RAV1 protein using the epitope NH_2_–CIDRQYSKKQRIVGAL–COOH as antigen, which is located at the C-terminal end of the PtRAV1 protein from *P. trichocarpa*. Antibodies were produced in rabbit and purified by Pineda Antikörper-Service (Berlin, Germany). The monospecific IgG fraction (in Tris–HCl buffer pH 7.5, 0.5 M NaCl, 1 mg/ml bovine serum albumin, 0.02% sodium azide) was 1:1 diluted with glycerol and stored at −20 °C.

### Protein extraction from stem tissues and Western immunoblotting

Basal branches were sampled in December 2012 and June 2013 to assess the expression of the transgenes in the field. About 250 mg of ground stem material were resuspended in 800 µl Laemmli sample buffer (61.9 mM Tris–HCl, 8 M urea, pH 6.8, 2% SDS), 5% β-mercaptoethanol, and 1X protease inhibitor mix for plant cell and tissue extracts (Sigma-Aldrich Co. LLC., Saint Louis, MO, USA). Tissue suspensions were vortexed for 1 min and sonicated in a water bath for 2 min, twice, and clarified by centrifugation for 15 min at 12,000*g* and room temperature. Proteins were precipitated overnight at 4 °C with 0.5 volumes of 50% trichloroacetic acid, and the following day were washed twice with 1 ml of cold acetone. Air-dried protein pellets were resuspended in 250 µl Laemmli sample buffer, 5% β-mercaptoethanol.

Proteins were separated on 10% sodium dodecyl sulfate-polyacrylamide gel electrophoresis (SDS-PAGE) gels and blotted onto 0.45 µm polyvinylidene difluoride membranes (Amersham™ Hybond™, GE Healthcare Life Sciences, Little Chalfont, UK). Immunoblottings were conducted as described previously [40] using a 1:1000 dilution of anti-haemagglutinin (anti-HA) (High-Affinity clone 3F1C; Roche Diagnostics, Indianapolis, IN, USA) or 1:500 of anti-PtRAV1 antibodies. Secondary hybridizations were run using a 1:100000 dilution of horseradish peroxidase (HRP)-linked goat anti-rabbit IgG (Sigma-Aldrich Co. LLC.). MagicMark™ XP Western Protein Standard (Thermo Fisher Scientific/Life Technologies/Invitrogen) was used as a molecular weight marker. Target proteins were detected using the Immobilon Western Chemiluminescent HRP Substrate (Merck Millipore, Billerica, MA, USA). To confirm equal loadings per lane, membranes were stained with Ponceau S.

### Growth-related and biomass measurements

Growth-related measurements for all trees in the trial were taken every year during dormancy periods (December 2012, 2013, 2014, and 2015). Heights (cm) of main stems and dominant shoots were measured using a pole. Diameters (mm) were measured over the bark at 130 cm above the ground using a digital caliper. Biomass yields were estimated by means of volumes and basal areas. Stems and dominant shoots volumes (cm^3^) were calculated from heights and diameters (at 10 cm above the ground) assuming a conical shape [*V* = 1/3(*πr* ^2^ *h*)]. Basal areas (cm^2^) (at 10 cm above the ground) were calculated as the circle area [*A* = *πr* ^2^] of each single stem in the first cultivation cycle, and as the sum of the circle areas of all shoots growing from each stump in the second cultivation cycle. Biomass yields were determined by recording the fresh weights of total aboveground biomass (stems and branches) per tree (kg) after the first (December 2013) and the second (December 2015) cultivation cycles. The fresh weight of each individual tree sampled in the field (weight accuracy of 50 g) was transformed into dry weight by estimating the moisture content at the genotype level. To do this, three complete trees (stems and branches) from each genotype and block, representing the most common diametric class, were oven-dried at 100 °C until constant weight.

### Wood chemistry and higher calorific value

After coppicing, 2-cm-thick main stem cross sections taken at 150, 200, and 250 cm above the ground were sampled from WT, CsRAV1 OX#60, and PtaRAV1&2 KD#1 trees (four trees per genotype, *n* = 4). Once the bark and pith were removed from the xylem, the disks were oven-dried for 48 h at 60 °C. Samples were ground in an ultra-centrifugal mill (RETSCH GmbH, Haan, Germany) until passing through a 0.75-mm sieve. Milled samples were sequentially extracted with dichloromethane (6 h), 95% ethanol (16 h), and distilled water (16 h). Extractions were run in a 125 ml Soxhlet apparatus on eleven batches of six samples (1.5 g per sample) keeping individuals separate in filter bags (ANKON Technology, Macedon, NY, USA). Extractive contents were determined by assessing weight loss after each step [41]. Klason lignin contents were determined in extractive-free samples following the procedure described by [42]. For analytical pyrolysis, about 30 mg of extracted samples were further milled in a vibratory ball mill (RETSCH GmbH) for 5 min, and stored in a desiccator. Pyrolysis analyses were performed using a Pyroprobe 1000 (CDS Analytical Inc, Oxford, PA, USA) with a coil filament connected to a gas chromatograph Agilent/HP7820 (Agilent Technologies Inc, Santa Clara, CA, USA) equipped with a flame ionization detector. Pyrolysis runs were conducted at 600 °C for 5 s on 75–82 µg of extractive-free ball-milled samples, and the resulting products were separated on a 60 m DB-1701 column (Agilent Technologies Inc). The syringyl/guaiacyl ratio (S/G) was calculated with ChemStation Software (Agilent Technologies, Palo Alto, USA) as the ratio of the sum of the areas of the S peaks divided by the sum of the area of G peaks. The pentosans/hexosans ratio (cP/cH) was calculated as the ratio of the sum of the areas of the pentosans peaks divided by the sum of the areas of hexosans peaks. The relative percentage of levoglucosan was calculated as the area of the levoglucosan peak relative to the sum of all identified peaks. Details about the conditions and quantification procedures have been published elsewhere [41, 43, 44].

The higher calorific value of the wood was established using the method outlined in International Standard ISO 1716. Three trees per genotype WT, CsRAV1 OX#60, and PtaRAV1&2 KD#1 were randomly selected. A representative wood sample per tree was ground in a mill (IKA^®^-Werke GmbH & CO. KG, Staufen, Germany) to a particle size of 0.5 mm. Pellets of about 1 g were prepared from the ground material using a hand press, oven-dried at 100 ± 5 °C for 24 h, and then weighed. Measurements were made using an adiabatic bomb calorimeter with a platinum resistance sensor PT-100 (IKA^®^-Werke GmbH & CO. KG). Higher calorific values were expressed as the average of measurements made in three pellets per tree.

### Histochemistry

Fifth internodes of several branches were collected in spring 2013. The sampled part of the branches was in the upright position and we took the samples from the zone corresponding to the side of the branch facing the main stem. We fixed the samples under vacuum in a solution of 4% formaldehyde (freshly prepared from paraformaldehyde) in phosphate-buffered saline (PBS: 137 mM NaCl, 0.27 mM KCl, 1 mM phosphate buffer, pH 7.4), kept overnight at 4 °C and then stored in a solution of 0.1% formaldehyde in PBS at 4 °C until further use. 50-µm-thick sections were cut on a Vibratome 1000 Plus (The Vibratome Company, St. Louis, MO, USA) under water. The sections were either stained with Calcofluor white to visualize cellulose or left untreated to detect lignin autofluorescence. Stacks of sections were collected on a confocal microscope Leica TCS SP8 (Leica Microsystems, Wetzlar, Germany) under the excitation line of 405 nm. Xylem areas were identified on the inner sides of the cambium cell layers, along with sclerenchyma-supporting tissue and cortex.

### Statistical analysis

A fixed-effect one-way ANOVA was used to assess differences in variables among genotypes. The linear model was

$$y_{ijk} = \mu + \beta_{i} + \delta_{j} + \varepsilon_{ijk},$$

where $y_{ijk}$ is the response of *k*th plant in the *j*th block of the *i*th event; $\mu$ is the overall mean; $\beta_{i}$ is the *i*th event effect; $\delta_{j}$ is the *j*th block effect, and $\varepsilon_{ijk}$ is the experimental error, $\varepsilon i_{jk} \sim N(0,\sigma^{2} )$.

All statistical analyses were carried out with R. The Shapiro–Wilk test was used to check the normality of the data and Levene test [45] to check the homoscedasticity. Normality was tested both for variable and residual distributions. When any of these assumptions was violated, the Kruskal–Wallis test [46] was used to analyze the data. To identify the differences among genotypes, we used the Tukey HSD post hoc test for ANOVA analyses and pairwise comparisons using the Wilcoxon test for Kruskal–Wallis analyses. The particular test used on each variable (trait) is detailed in Additional file 1: Table S1.

## Results

### Sylleptic branching and genetic modifications are retained over cultivation cycles

**Fig. 1:**
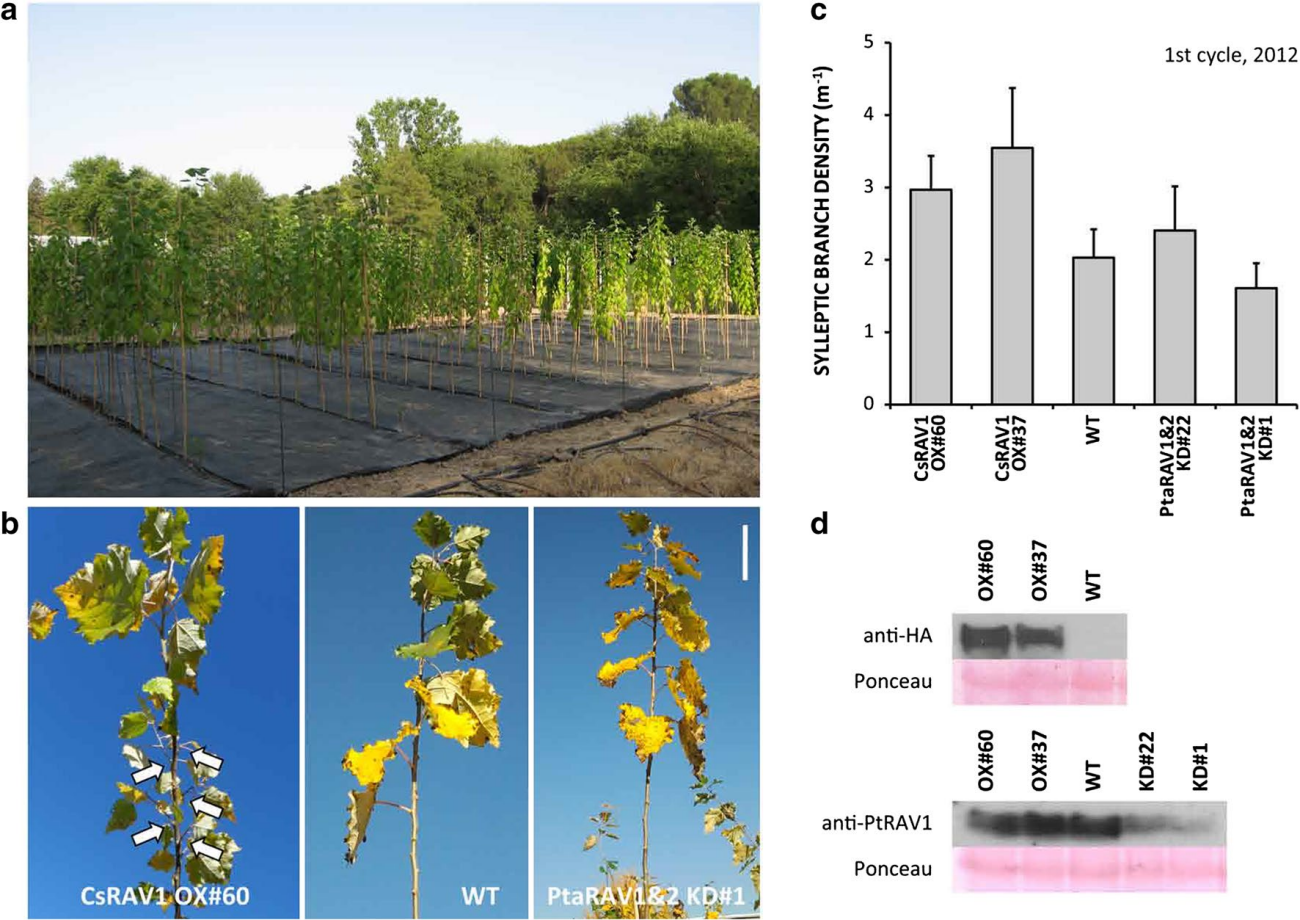
Field trial establishment, syllepsis of RAV1-engineered poplars and RAV1-protein abundances during the first cultivation cycle. **a** Image of the field trial once established (July 2012). **b** Sylleptic branches on the apical segment of the main stem in the representative event CsRAV1 OX#60 (*white arrows*), as opposed to wild-type (WT), and event PtaRAV1&2 KD#1 (November 2012); *bar* 10 cm. **c** Densities of sylleptic branches on the main stem of WT and CsRAV1-overexpression and PtaRAV1&2-knockdown transgenic poplars at the end of the establishment year (December 2012). *Bars* represent average values ± SE (CsRAV1 OX#60 *n* = 30, CsRAV1 OX#37 *n* = 30, WT *n* = 29, PtaRAV1&2 KD#22 *n* = 25, PtaRAV1&2 KD#1 *n* = 30). **d** *Upper panel* Western blot of the chestnut transgenic protein CsRAV1 tagged to 3xHA in both CsRAV1-overexpression events tested and the WT. *Lower panel* Western blot of the poplar endogenous protein PtaRAV1 in all four transgenics and the WT as control. Membranes were stained with Ponceau to ensure equal sample loading

The present field trial was established in July 2012 in an experimental plot in Madrid (Spain), and included 30 trees per genotype distributed in 3 blocks (Fig. 1a). That year, during the remaining growing season, the occurrence of sylleptic branches in CsRAV1-overexpression poplars was evident (Fig. 1b). In December 2012, average densities of sylleptic branches (i.e., number of branches per unit of stem length) in CsRAV1-overexpressors tended to be about 50% (event #60) and 75% (event #37) higher than in wild-type (WT) trees (*p* > 0.05) (Fig. 1c; see Additional file 1: Table S1 and Additional file 2: Figure S1a). During the next growing season of this first cultivation cycle, axillary buds on the new growth of the main stem or on lateral branches, both sylleptics and proleptics, did not burst in any of the five genotypes on trial. A major concern about the sustainability of genetically modified crops is related to the potential instability of the introduced genetic modification over time, involving silencing mechanisms that could disable the desired trait [37]. To test whether the introduced genetic modifications persisted over time, during 2013 the basal branches were sampled to analyze the stability of those transgenes in the field. The transgenic fusion protein 3xHA:CsRAV1 was detected in both CsRAV1 OX events, whereas the endogenous target protein PtaRAV1 was detected in all transgenic and WT trees, showing a similar abundance in CsRAV1-overexpressors and WT trees, and very reduced levels in PtaRAV1&2 KD events #1 and #22 relative to the WT (Fig. 1d). It indicated that the genetic modifications introduced in these poplars, CsRAV1 overexpression and PtaRAV1 downregulation, continued functioning after several months of growing in the field, and that both events tested per modification behaved similarly at the molecular level.

**Fig. 2:**
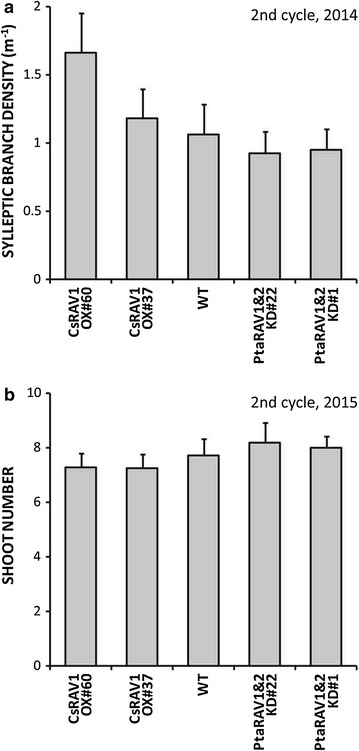
Sylleptic branching and shoot resprouting phenotypes of RAV1-engineered poplars during the second cultivation cycle. **a** Densities of sylleptic branches on the dominant shoots of wild-type (WT) and CsRAV1-overexpression and PtaRAV1&2-knockdown transgenics. Measurements were made in December 2014 at the end of the first growing season after the first coppicing. **b** Shoot number growing from the remaining 10-cm-long stumps of WT and events CsRAV1 OX and PtaRAV1&2 KD. Scoring was made before a second harvest in December 2015.* Bars* represent average values ± SE (CsRAV1 OX#60 *n* = 30, CsRAV1 OX#37 *n* = 30, WT *n* = 29, PtaRAV1&2 KD#22 *n* = 30, PtaRAV1&2 KD#1 *n* = 30)

After coppicing in December 2013, trees grew as multi-trunk individuals with multiple shoots resprouting from the remaining 10-cm-long stumps. As in the first cultivation cycle, sylleptic branches developed during the first but not the second growing season of the cycle. So, at the end of 2014, we calculated densities of sylleptic branches growing along dominant shoots (i.e., the highest and thickest shoot resprouted from each tree stump). Average densities of sylleptics on dominant shoots in both CsRAV1 OXs tended to be higher, about 9% (event #37) and 55% (event #60) higher than in WT trees. Conversely, PtaRAV1&2 KDs developed some 10% (event #1) and 18% (event #22) less sylleptics than WT trees (*p* > 0.05) (Fig. 2a; see Additional file 1: Table S1 and Additional file 2: Figure S1a). CsRAV1 OX and PtaRAV1&2 KD events showed a greater and a slightly lower degree of syllepsis, respectively, relative to WT trees. This tendency, which persisted up until the completion of the field trial 4 years after its establishment, suggested that those genetic modifications were working over all that time.

Shoots growing from each coppiced tree stump were also counted. Data were collected in December 2015, on completion of the second cultivation cycle, and they revealed that CsRAV1 OX and PtaRAV1&2 KD events tended to develop slightly fewer (c. 5%) and more (c. 5%) shoots, respectively, relative to WT trees (*p* > 0.05) (Fig. 2b; see Additional file 1: Table S1 and Additional file 2: Figure S1b).

### Genetically modified trees maintained the same structural, chemical composition, and combustion wood properties as the WT poplars

**Fig. 3:**
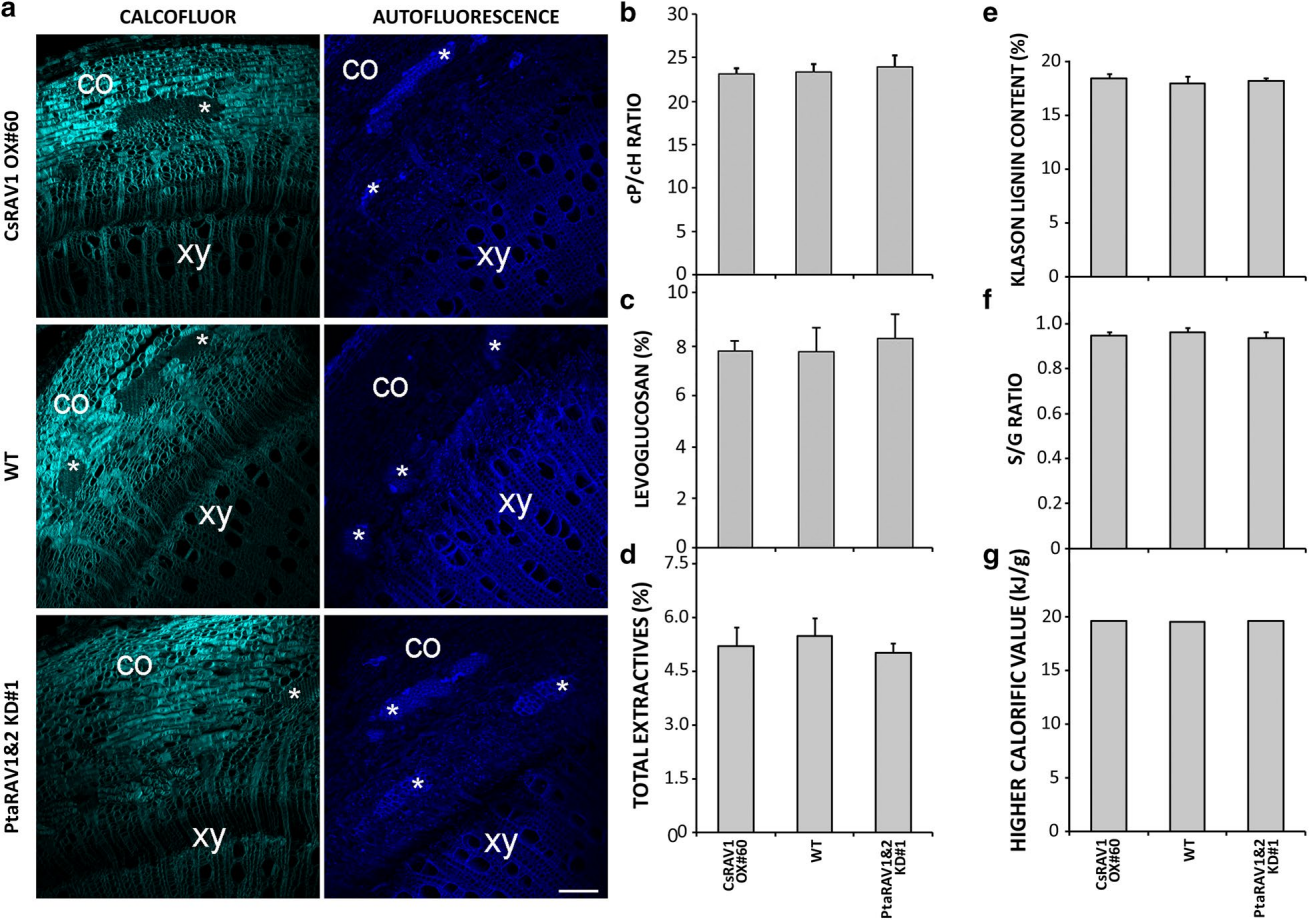
Wood structure and chemical wood composition of the RAV1-engineered poplars. **a** Wood histochemistry analyses of branch cross sections (5th internode) obtained from wild-type (WT) trees and representative events 3xHA:CsRAV1 OX#60 and PtaRAV1&2 KD#1. The sections, taken from the side of branches facing the main stem, were sampled after coppicing in December 2013. *Left column* cellulose detection by Calcofluor white staining. *Right column* detection of lignin autofluorescence. *co* cortex, *xy* xylem, * *sclerenchyma bar* 100 μm. **b**–**f** Xylem composition of WT trees and representative events CsRAV1 OX#60 and PtaRAV1&2 KD#1 after coppicing in December 2013, including cP/cH ratio and percentage of levoglucosan, total extractives, Klason lignin content, S/G ratio.* Bars* represent average values ± SD (CsRAV1 OX#60 *n* = 4, WT *n* = 4, PtaRAV1&2 KD#1 *n* = 4). **g** Higher calorific values of coppiced biomass obtained from WT trees and events CsRAV1 OX#60 and PtaRAV1&2 KD#1.* Bars* represent average values ± SD (CsRAV1 OX#60 *n* = 3, WT *n* = 3, PtaRAV1&2 KD#1 *n* = 3)

Besides transgene stability over time, another major concern about transgenesis is pleiotropy and non-desirable side effects caused by the introduced genetic change. The assayed transgenics in this field trial showed an unaltered overall health condition with respect to the WT trees. Closer inspection was made of those traits concerning the quality of the produced wood. Individuals of CsRAV1 OX#60, WT, and PtaRAV1&2 KD#1 tree genotypes were randomly selected to compare anatomy, chemical composition, and combustion properties of their woods. Calcofluor white staining and lignin autofluorescence of branch sections (fifth internodes) showed a similar overall structure and organization, as well as similar cellulose and lignin contents of the transgenic and WT woods (Fig. 3a). Chemical analyses confirmed that there were no significant differences among these genotypes in wood extractives (*p* > 0.05), Klason lignin contents (*p* > 0.05), syringyl/guaiacyl (S/G) subunit ratios (*p* > 0.05), pentosans/hexosans (cP/Ch) (*p* > 0.05), and levoglucosan (*p* > 0.05) (Fig. 3b–f; see Additional file 1: Table S1). We further determined wood higher calorific values for these genotypes, and in accordance with the ascertained data for wood composition found that the transgenic and WT woods produced the same amount of heat by combustion (*p* > 0.05) (Fig. 3g; see Additional file 1: Table S1). Thus, it is reasonable to predict that any modification of the RAV1 gene expression in a commercial elite poplar clone is not likely to affect the structure and composition of its wood, nor the bioenergy properties of its biomass.

### RAV1-engineering impacts differentially on growth and aerial biomass yield over cultivation cycles

**Fig. 4:**
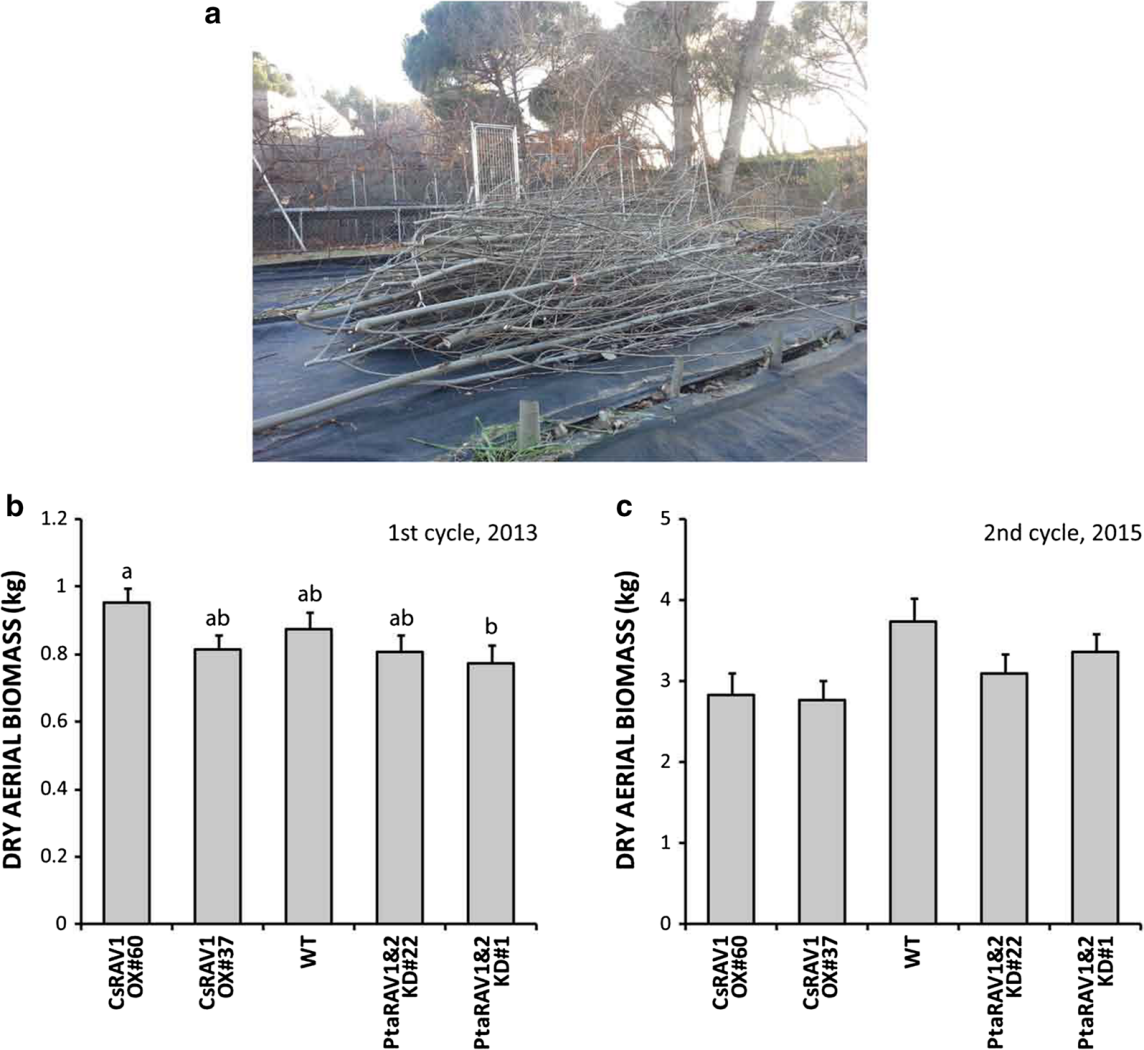
Aboveground biomass yields of the RAV1-engineered poplars after two cultivation cycles. **a** Picture of the field trial after coppicing in December 2013, showing the 10-cm-long stumps. Dry aerial biomass yields of wild-type (WT) and CsRAV1-overexpression and PtaRAV1&2-knockdown transgenics, after **b** the first coppicing in December 2013, and **c** the second coppicing in December 2015. *Bars* represent average values ± SE (CsRAV1 OX#60 *n* = 30, CsRAV1 OX#37 *n* = 30, WT *n* = 29, PtaRAV1&2 KD#22 *n* = 30, PtaRAV1&2 KD#1 *n* = 30). *Letters* represent significant differences between genotypes (*p* < 0.05)

On completion of the first cultivation cycle in December 2013 (Fig. 4a), event CsRAV1 OX#60 showed an average diameter of its main stem about 6% thicker and an average aerial biomass yield about 9% greater than in WT trees. Conversely, event PtaRAV1&2 KD#1 displayed an average diameter of its main stem that was some 6% thinner and an average aerial biomass yield about 11% lower than in WT trees (stem diameter *p* < 0.01; aerial biomass yield *p* < 0.05) (Table 1; Fig. 4b; see Additional file 1: Table S1, Additional file 3: Figure S2a, Additional file 4: Figure S3). However, significance relied solely when comparing means from CsRAV1 OX#60 and PtaRAV1&2 KD#1 genotypes (stem diameter *p* < 0.05; aerial biomass yield *p* < 0.05). Estimators for biomass yield, both volume and basal area in the coppice year 2013/2015 appeared to be in accordance with the above growth and production results (Table 1; Additional file 1: Table S1 and Additional file 5: Figure S4) Therefore, these results obtained over the course of a first cultivation cycle (before coppicing) stand up for the viability of RAV1-engineering to improve aerial biomass yields of high-density poplar plantations of trees growing as single-trunk individuals.

**Table 1:** Summary of growth-related data recorded from RAV1-engineered poplars over the course of the field trial

	CsRAV1 OX#60	CsRAV1 OX#37	Wild-type	PtaRAV1&2 KD#22	PtaRAV1&2 KD#1
First rotation					
Year 2012					
Stem height (cm)	321.3 ± 5.0a	313.8 ± 3.7ab	318.1 ± 4.0a	295.2 ± 5.6b	313.9 ± 4.6ab
Stem diameter (mm)	12.9 ± 0.4a	12.8 ± 0.3a	11.9 ± 0.3ab	10.7 ± 0.4b	11.8 ± 0.3ab
Stem volume (cm^3^)	413.5 ± 26.2ns	381.7 ± 20.3ns	376.1 ± 25.4ns	317.4 ± 24.4ns	383.5 ± 25.2ns
Basal area (cm^2^)	3.8 ± 0.2ns	3.6 ± 0.2ns	3.5 ± 0.2ns	3.1 ± 0.2ns	3.6 ± 0.2ns
Year 2013					
Stem height (cm)	506.8 ± 11.1ns	484.8 ± 9.2ns	498.8 ± 12.7ns	496.1 ± 11.5ns	475.7 ± 15.4ns
Stem diameter (mm)	24.8 ± 0.6a	23.1 ± 0.5ab	23.3 ± 0.6ab	22.4 ± 0.6ab	21.5 ± 0.8b
Stem volume (cm^3^)	2090.3 ± 111.2ns	1747.1 ± 120.1ns	1855.7 ± 129.2ns	1790.9 ± 118.6ns	1589.7 ± 123.8ns
Basal area (cm^2^)	12.2 ± 0.5a	10.6 ± 0.6ab	10.9 ± 0.6ab	10.7 ± 0.6ab	9.7 ± 0.6b
Second rotation					
Year 2014					
Dominant shoot height (cm)	537.9 ± 11.9bc	515.6 ± 11.5b	602.3 ± 10.1a	565.4 ± 9.8ac	574.8 ± 9.1ac
Dominant shoot diameter (mm)	21.1 ± 0.9a	20.6 ± 0.8a	26.7 ± 0.8b	24.6 ± 1.0b	26.4 ± 0.8b
Dominant shoot volume (cm^3^)	1713.7 ± 155.8ns	1515.6 ± 138.6ns	2444.5 ± 170.1ns	1936.1 ± 131.5ns	2274.6 ± 146.6ns
Basal area (cm^2^)	82.7 ± 9.2ns	88.4 ± 12.4ns	92.7 ± 12.6ns	104.9 ± 15.8ns	94.1 ± 10.7ns
Year 2015					
Dominant shoot height (cm)	704.1 ± 20.0a	679.5 ± 20.5a	793.5 ± 16.9b	728.2 ± 21.0ab	779.7 ± 15.5b
Dominant shoot diameter (mm)	31.2 ± 1.3a	30.0 ± 1.2a	36.6 ± 1.3b	33.3 ± 1.3ab	35.9 ± 1.0b
Dominant shoot volume (cm^3^)	3818.1 ± 389.6ns	3291.2 ± 303.9ns	4809.4 ± 402.4ns	3972.7 ± 359.9ns	4736.5 ± 319.0ns
Basal area (cm^2^)	124.9 ± 26.0ns	106.8 ± 13.8ns	109.5 ± 15.3ns	119.2 ± 16.8ns	108.1 ± 12.1ns

Average values for heights and diameters of the main stem and the dominant shoot, and for biomass yield estimators volumes and basal areas ± SE (CsRAV1 OX#60 *n* = 30, CsRAV1 OX#37 *n* = 30, WT *n* = 29, PtaRAV1&2 KD#22 *n* = 30, PtaRAV1&2 KD#1 *n* = 30) of wild-type (WT) and CsRAV1-overexpression and PtaRAV1&2-knockdown transgenics measurements were made at the end of every year. Letters a, b, and c represent significant differences between genotypes (*p* < 0.05)

*ns* no significance

On completion of the second cultivation cycle in December 2015, shoot growth and aerial biomass yields data from the CsRAV1 OX events revealed that despite having developed sylleptic branches, dominant shoots from both CsRAV1-overexpressors were smaller than in WT trees, showing reduced average diameters (*p* < 0.001) and heights (*p* < 0.001). Diameters were reduced to about 15% (event #60 *p* < 0.05) and 18% (event #37 *p* < 0.01); heights were reduced to about 11% (event #60 *p* < 0.05) and 14% (event #37 *p* < 0.01) (Table 1; see Additional file 1: Table S1 and Additional file 3: Figure S2b). As a result, these transgenics tended to yield an average aerial biomass that was some 25% less than in WT trees (*p* > 0.05). Unexpectedly, growth performance of PtaRAV1&2-knockdown events was slightly altered, leading them to show a downward trend in their yields, about 10% (event #1) and 17% (event #22) less aerial biomass than WT trees (*p* > 0.05) (Fig. 4c; see Additional file 4: Table S1 and Additional file 4: Figure S3). Their dominant shoots tended to display reduced average diameters and heights of about 5% for both traits (*p* > 0.05) (Table 1; see Additional file 4: Table S1 and Additional file 3: Figure S2b).

## Discussion

Cultivation of poplar and other fast-growing woody species as SRC is an increasingly widespread practice for the production of lignocellulosic biomass as carbon–neutral renewable energy source. Productivity and sustainability of forest and SRC plantations depend on the cultivars used and also and very importantly on the interactions with the environmental conditions over time. In this work, we established a field trial to test the sustainability of the increased sylleptic branchiness of CsRAV1-overexpression hybrid poplars over two subsequent cultivation cycles and its impact on biomass production. In outdoors conditions, those interactions are much more complex than in a greenhouse and therefore plants may show a greater phenotypic variation, making unpredictable the outcome of such experimental approach.

On completion of the first cultivation cycle in December 2013, aerial biomass yields and stem growth data from events CsRAV1 OX#60 and PtaRAV1&2 KD#1 were consistent with those reported in other studies, in which sylleptics were noted to contribute to the thickening of stems by allocating to a greater portion of photosynthates than proleptics, and hence to enhance the aboveground biomass yield [16–21]. On completion of the second cultivation cycle in December 2015, aerial biomass yields and shoot growth data from CsRAV1-overexpressors pointed to what has been reported for the relationship between syllepsis and stem growth and its dependency on the genetic material and on the environmental conditions [17, 21]. Despite tending to develop more sylleptic branches, dominant shoots from both events CsRAV1 OX were smaller than in WT trees. Also, shoot resprouting after coppicing was slightly reduced in these events, suggesting that the available nutrient resources were preferably invested in the production of sylleptics. Conversely, both events PtaRAV1&2 KD favored the production of few sylleptics and more resprouts than WT trees, but still their dominant shoots tended to be smaller than in WT trees. As reported with syllepsis, number of shoots is also positively correlated with aboveground biomass yield [21]. In this field trial, any modification of RAV1 expression somehow reduced, to a greater (in CsRAV1 OX trees) or lesser (in PtaRAV1&2 KD trees) extent, dimensions of dominant shoots, which was ultimately translated into a loss of aerial biomass. The multigenic nature of the biomass yield and related traits and the complex phenotypic and genotypic relationships existing among them, as well as the important effect of the environmental conditions on their expression over time, might be the underlying causes of the results described above.

We concluded that over the course of two cultivation cycles, CsRAV1-overexpression hybrid poplars tended to show an enhanced development of sylleptic branching in the field. This fact confirmed that local geoclimate factors and the chosen culture conditions of planting density, watering, and fertilization regimes were adequate to allow for and sustain syllepsis in CsRAV1-overexpression poplars, at least during the first growing seasons of each cultivation cycle as single- and multi-trunk individuals (first and second cultivation cycles, respectively). Several studies on poplar crown architecture (i.e., the branching pattern) have been carried out due to its great impact on biomass productivity: it determines leaf orientation and distribution, canopy density, light interception, and hence carbon assimilation. Branching habit of a specific genotype relayed on their genetic features and underlying hormonal and physiological mechanisms [20, 29, 47–50]. An extensive study by Broeck et al. [20] on the crown architecture in SRC plantation with four poplar commercial genotypes found significant differences in their ability to sustain sylleptic branches during the second growing season (absent in genotype Wolterson and enhanced in genotype Koster). In our field trial, the absence of syllepsis during the second seasons could be due to the hybrid poplar clone used (to our knowledge no information about its branching habit is publicly available). However, we cannot discard the influence of exogenous factors such as larger shading during the second growing seasons than during the first ones [23].

It is worth noting that average amounts of aerial biomass obtained from the hybrid poplars used in this trial, widely used in basic research, were far from those reported for commercial poplar varieties bred to produce good yields [25], so the viability of RAV1-engineering will depend on the genetic transformation of these commercial elite trees. In addition, disparity of results between the events of the same transgenic line (CsRAV1-overexpression line or PtaRAV1&2-knockdown line) points out the necessity and importance of selecting the best performing events in the field.

## Conclusions

In summary, measurements made during a first cultivation cycle on single-trunk trees showed that, in addition to early branching, biomass yields could be enhanced at least in one field-assayed CsRAV1-overexpression event. These findings support the potential application of CsRAV1-overexpression to increase syllepsis in commercial elite trees without changing wood quality. During a second cultivation cycle, both field-assayed CsRAV1-overexpression events growing as multi-trunk trees showed reduced aerial growth and biomass yields compared to the control. Yet, improvements on syllepsis development were maintained in this second cultivation cycle, which represents a significant step forward in translating valuable traits from the laboratory to the field, where they must be tested. Thus, RAV1-engineering or marker-assisted breeding based on this gene followed by the selection of the best performing events or individuals could certainly help to improve early branching of hybrid poplar in SRC. However, the eventual goal to sustain and enhance biomass productivity through the modification of the expression of this gene will depend on future developments. A better understanding of the genetic basis of a complex phenotype such as biomass productivity and its potential determinants is needed, as well as a deeper knowledge of the phenotypic and genetic relationships among biomass-related traits and how these relationships are affected by the environment. In this context, tree biotechnology has the potential to provide a means to develop forest plantations highly productive and sustainable, which in turn will help conserve natural forests and mitigate the effects of climate change. Indeed, few other options can match the potential of forestry in this respect [51].

## Additional files

**Additional file 1: Table S1.** Statistical tests used to analyze all traits measured over the course of the field trial. Differences among genotypes were identified using post hoc Tukey HSD test for ANOVA analyses, and pairwise comparisons with the Wilcoxon test for Kruskal–Wallis analyses.

**Additional file 2: Fig. S1.** Syllepsis and shoot resprouting performance of the RAV1-engineered poplars in the field. Scatterplots showing the distribution of individual values per block (a) for densities of sylleptic branches on the main stem (first cultivation cycle, upper graph) and on the dominant shoot (second cultivation cycle, lower graph); and (b) for the number of shoots resprouting from the remaining 10 cm-long stumps. Counting of sylleptic branches was made in December 2012 and 2014, and shoots in December 2015, respectively. Horizontal lines represent median values per block.

**Additional file 3: Fig. S2.**Growth-related characteristics of the RAV1-engineered poplars in the field. Scatterplots showing the distributions of individual values per block for heights and diameters (a) of the main stem (first cultivation cycle, years 2012 and 2013) and (b) of the dominant shoot (second cultivation cycle, years 2014 and 2015) of wild-type (WT) and CsRAV1-overexpressing and PtaRAV1&2-knockdown transgenic poplars. Horizontal lines represent median values per block.

**Additional file 4: Fig. S3.**Aboveground biomass yields of the RAV1-engineered poplars after two cultivation cycles. Scatterplots showing the distributions of individual values per block, for the aerial biomass production of wild-type (WT) and CsRAV1-overexpression and PtaRAV1&2-knockdown transgenics. Trees were coppiced in December 2013 (first cultivation cycle, upper graph) and December 2015 (second cultivation cycle, lower graph). Horizontal lines represent median values per block.

**Additional file 5: Fig. S4.**Stem volume and basal area of the RAV1-engineered poplars after two cultivation cycles. Scatterplots showing the distributions of individual values per block, for the stem volume and basal area of wild-type (WT) and CsRAV1-overexpression and PtaRAV1&2-knockdown transgenics. Trees were coppiced in December 2013 (first cultivation cycle, upper graph) (a) and December 2015 (second cultivation cycle, lower graph) (b). Horizontal lines represent median values per block.

## Abbreviations

co: cortex
DNA: deoxyribonucleic acid
G: guaiacyl
GWAS: genome-wide association study
HA: haemagglutinin
hpiRNA: hairpin RNA interference
IgG: immunoglobulin G
KD: knockdown
OX: overexpression
RNA: ribonucleic acid
S: syringyl
SDS-PAGE: sodium dodecyl sulfate polyacrylamide gel electrophoresis
SRC: short-rotation coppice
WT: wild-type
xy: xylem
