# Supplementary material for: Impact of RAV1-engineering on poplar biomass production: a short-rotation coppice field trial
Source: Biotechnol Biofuels. 2017 May 2;10:110. doi: 10.1186/s13068-017-0795-z (PMC5414296; doi:10.1186/s13068-017-0795-z)
Supplement: Supplementary file 1 — Additional file 1: Table S1. Statistical tests used to analyze all traits measured over the course of the field trial. Differences among genotypes were identified using post hoc Tukey HSD test for ANOVA analyses, and pairwise comparisons with the Wilcoxon test for Kruskal–Wallis analyses. [file 13068_2017_795_MOESM1_ESM.docx]

**Table S1. Statistical tests used to analyze all traits measured over the course of the field trial.**

|  | ***Year*** | ***Trait*** | ***One-way analysis of variance test*** |
| --- | --- | --- | --- |
| ***First cultivation cycle*** | ***2012*** | *Sylleptic branch density* | Kruskal-Wallis |
|  |  | *Stem height* | Kruskal-Wallis |
|  |  | *Stem diameter* | Kruskal-Wallis |
|  |  | *Stem volumen* | Kruskal-Wallis |
|  |  | *Basal area* | Kruskal-Wallis |
|  | ***2013*** | *Stem height* | Kruskal-Wallis |
|  |  | *Stem diameter* | Kruskal-Wallis |
|  |  | *Dry aerial biomass* | ANOVA |
|  |  | *Total extractives* | ANOVA |
|  |  | *cP/cH ratio* | ANOVA |
|  |  | *Levoglucosan* | ANOVA |
|  |  | *Klason lignin content* | ANOVA |
|  |  | *S/G ratio* | ANOVA |
|  |  | *Higher calorific value* | ANOVA |
|  |  | *Stem volumen* | Kruskal-Wallis |
|  |  | *Basal area* | Kruskal-Wallis |
| ***Second cultivation cycle*** | ***2014*** | *Sylleptic branch density* | Kruskal-Wallis |
|  |  | *Dominant shoot height* | Kruskal-Wallis |
|  |  | *Dominant shoot diameter* | ANOVA |
|  |  | *Stem volumen* | Kruskal-Wallis |
|  |  | *Basal area* | Kruskal-Wallis |
|  | ***2015*** | *Shoot number* | Kruskal-Wallis |
|  |  | *Dry aerial biomass* | Kruskal-Wallis |
|  |  | *Dominant shoot height* | Kruskal-Wallis |
|  |  | *Dominant shoot diameter* | ANOVA |
|  |  | *Stem volumen* | Kruskal-Wallis |
|  |  | *Basal area* | Kruskal-Wallis |

Differences among genotypes were identified using *post-hoc* Tukey HSD test for ANOVA analyses, and pairwise comparisons with the Wilcoxon test for Kruskal-Wallis analyses.
