# Supplementary material for: Impact of RAV1-engineering on poplar biomass production: a short-rotation coppice field trial
Source: Biotechnol Biofuels. 2017 May 2;10:110. doi: 10.1186/s13068-017-0795-z (PMC5414296; doi:10.1186/s13068-017-0795-z)
Supplement: Supplementary file 2 — Additional file 2: Fig. S1. Syllepsis and shoot resprouting performance of the RAV1-engineered poplars in the field. Scatterplots showing the distribution of individual values per block (a) for densities of sylleptic branches on the main stem (first cultivation cycle, upper graph) and on the dominant shoot (second cultivation cycle, lower graph); and (b) for the number of shoots resprouting from the remaining 10 cm-long stumps. Counting of sylleptic branches was made in December 2012 and 2014, and shoots in December 2015, respectively. Horizontal lines represent median values per block. [file 13068_2017_795_MOESM2_ESM.pdf]

Additional file 2

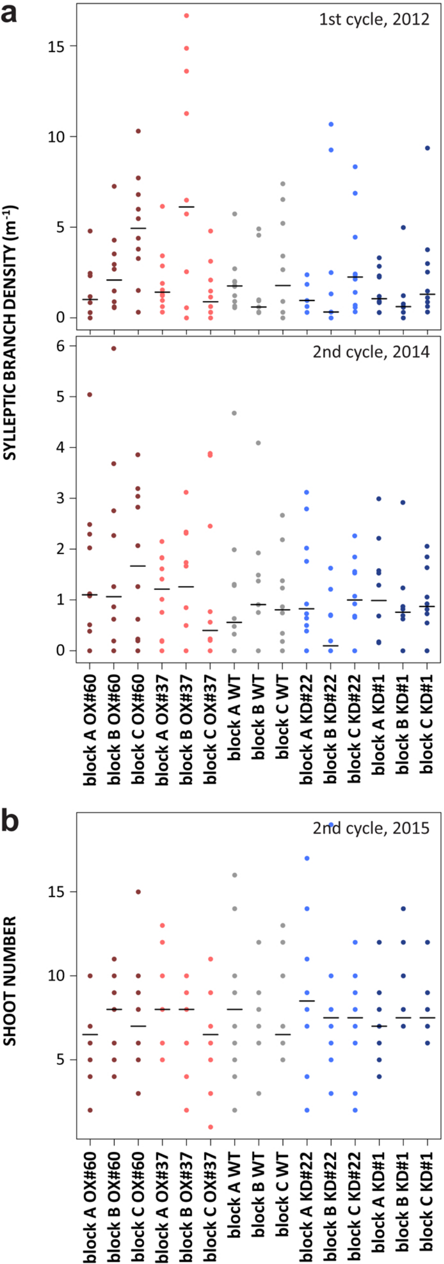

Figure S1. Syllepsis and shoot resprouting performance of the RAV1-engineered poplars in the field. Scatterplots showing the distribution of individual values per block (a) for densities of sylleptic branches on the main stem (first cultivation cycle, upper graph) and on the dominant shoot (second cultivation cycle, lower graph); and (b) for the number of shoots resprouting from the remaining 10 cm-long stumps. Counting of sylleptic branches was made in December 2012 and 2014, and shoots in December 2015, respectively. Horizontal lines represent median values per block.
