# Supplementary material for: Impact of RAV1-engineering on poplar biomass production: a short-rotation coppice field trial
Source: Biotechnol Biofuels. 2017 May 2;10:110. doi: 10.1186/s13068-017-0795-z (PMC5414296; doi:10.1186/s13068-017-0795-z)
Supplement: Supplementary file 3 — Additional file 3: Fig. S2.Growth-related characteristics of the RAV1-engineered poplars in the field. Scatterplots showing the distributions of individual values per block for heights and diameters (a) of the main stem (first cultivation cycle, years 2012 and 2013) and (b) of the dominant shoot (second cultivation cycle, years 2014 and 2015) of wild-type (WT) and CsRAV1-overexpressing and PtaRAV1&2-knockdown transgenic poplars. Horizontal lines represent median values per block. [file 13068_2017_795_MOESM3_ESM.pdf]

Additional file 3

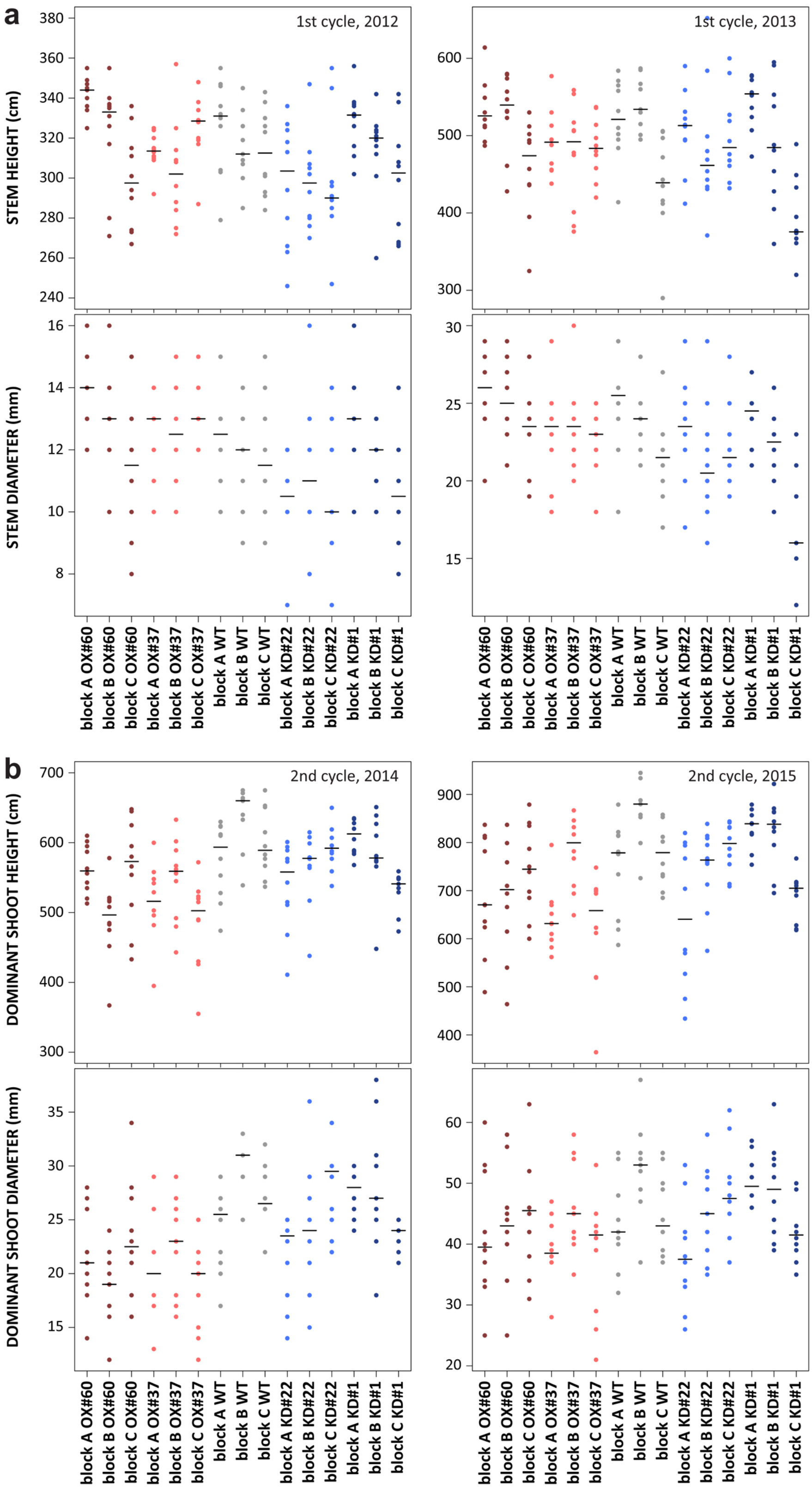

Figure S2. Growth-related characteristics of the RAV1-engineered poplars in the field. Scatterplots showing the distributions of individual values per block (a) for heights and diameters (a) of the main stem (first cultivation cycle, years 2012 and 2013) and (b) of the dominant shoot (second cultivation cycle, years 2014 and 2015) of wild-type (WT) and CsRAV1-overexpressing and PtaRAV1&2-knockdown transgenic poplars. Horizontal lines represent median values per block.
