# Supplementary material for: Impact of RAV1-engineering on poplar biomass production: a short-rotation coppice field trial
Source: Biotechnol Biofuels. 2017 May 2;10:110. doi: 10.1186/s13068-017-0795-z (PMC5414296; doi:10.1186/s13068-017-0795-z)
Supplement: Supplementary file 5 — Additional file 5: Fig. S4.Stem volume and basal area of the RAV1-engineered poplars after two cultivation cycles. Scatterplots showing the distributions of individual values per block, for the stem volume and basal area of wild-type (WT) and CsRAV1-overexpression and PtaRAV1&2-knockdown transgenics. Trees were coppiced in December 2013 (first cultivation cycle, upper graph) (a) and December 2015 (second cultivation cycle, lower graph) (b). Horizontal lines represent median values per block. [file 13068_2017_795_MOESM5_ESM.pdf]

Additional file 5

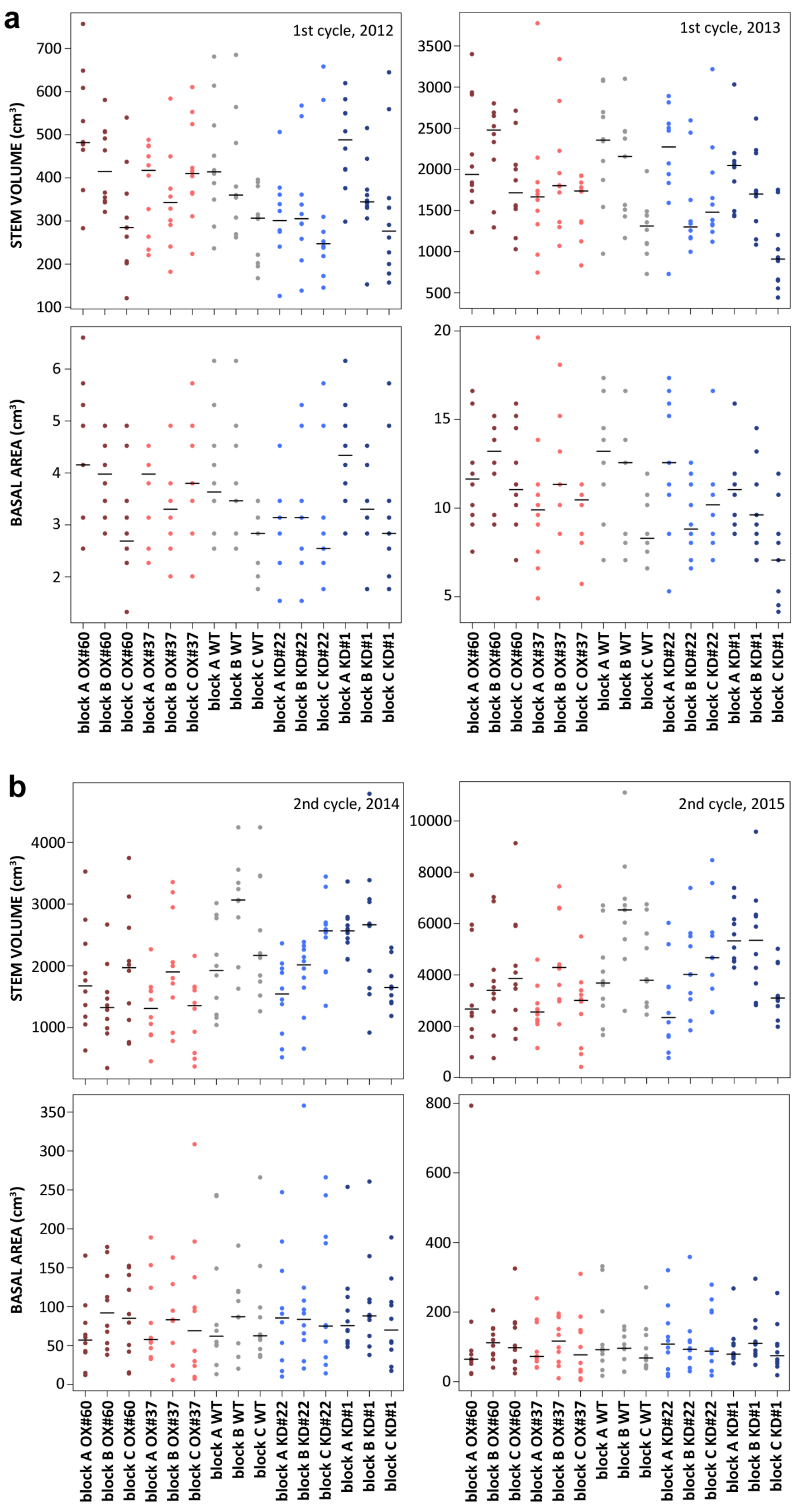

Figure S4. Stem volume and basal area of the RAV1-engineered poplars after two cultivation cycles. Scatterplots showing the distributions of individual values per block, for the stem volume and basal area of wild-type (WT) and CsRAV1-overexpression and PtaRAV1&2-knockdown transgenics. Trees were coppiced in December 2013 (first cultivation cycle, upper graph) (a) and December 2015 (second cultivation cycle, lower graph) (b). Horizontal lines represent median values per block.
